# Cognitive Profiles in Alzheimer's Disease: Insights from a Multicenter Study in Peru

**DOI:** 10.1002/alz70857_107776

**Published:** 2025-12-25

**Authors:** Jonathan Adrian Zegarra‐Valdivia, Milagros del Rocio Casimiro‐Arana, Leandro Aron Perez‐Fernandez, Harold Alessandro Arana‐Nombera, Viviana Nayelli Gallegos‐Manayay, María del Rosario Oliva‐Piscoya, Reyna Elizabeth Alamo‐Medina, Leslie Magdalena Lozada Tantarico, Brenda Nadia Chino‐Vilca, Carmen Paredes Manrique, Jose Carreño‐Galvez, Jose Cabrejo, Ernesto Gabriel Adolfo‐Espinoza, Emily Gonzales‐Manayalle, Diana Gutierrez‐Flores, Gilda Flores‐Valdivia, Noelia Graciela Raffo‐Moron, César Ramal‐Ansayac, Maria Celinda Cruz Ordinola, Nilton Custodio, Sheila Castro‐Suarez

**Affiliations:** ^1^ Universidad Señor de Sipán, Chiclayo, Peru; ^2^ Universidad San Martín de Porres, Chiclayo, Peru; ^3^ Achucarro Basque Center for Neuroscience, Bilbao, Spain; ^4^ Universidad Tecnológica del Perú, Arequipa, Peru; ^5^ Universidad Peruana Unión, filial Juliaca, Puno, Perú, Puno, Peru; ^6^ Hospital Almanzor Aguinaga Asenjo, Chiclayo, Lambayeque, Peru; ^7^ MINSA, Lima, Lima, Peru; ^8^ Universidad Señor de Sipan, Chiclayo, Lambayeque, Peru; ^9^ Universidad Continental, Arequipa, Arequipa, Peru; ^10^ Universidad continental de Arequipa, Arequipa, Arequipa, Peru; ^11^ GERESA, Iquitos, Loreto, Peru; ^12^ Unit Cognitive Impairment and Dementia Prevention, Peruvian Institute of Neurosciences, Lima, Peru, Lima, Lima, Peru; ^13^ Research unit, Instituto Peruano de Neurociencias, Lima, Peru; ^14^ Atlantic Fellow for Equity in Brain Health at Global Brain Health Institute (GBHI), San Francisco, CA, Peru; ^15^ CBI en Demencias y Enfermedades Desmielinizantes del Sistema Nervioso, Instituto Nacional de Ciencias Neurológicas, Lima, Peru

## Abstract

**Background:**

Mild cognitive impairment (MCI) and Alzheimer's disease (AD) are progressive neurodegenerative conditions affecting cognition and daily functioning. In Peru, this is the first multi‐site study conducted across Lima, Arequipa, Puno, Chiclayo, and Loreto to evaluate cognitive performance using standardized neuropsychological instruments. The objective was to characterize participants with MCI and AD through a comprehensive neuropsychological assessment.

**Method:**

A total of 267 patients were evaluated, including 145 controls, 41 individuals with MCI, and 81 with AD. Standardized assessments included PHQ‐9, Pfeffer, RUDAS, and INECO. RUDAS and INECO scores were compared between groups using appropriate statistical tests (T‐Student or Mann‐Whitney U).

**Result:**

AD patients exhibited significant cognitive and executive impairments compared to controls.

RUDAS Total Score: AD (11.26 ± 8.3) vs. Controls (26.61 ± 3.1, *p* < 0.001). INECO Total Score: AD (1.01 ± 3.29) vs. Controls (1.32 ± 4.93, *p* =  0.363, Mann‐Whitney U). The most affected INECO subtests in AD patients included Instrucciones Conflictivas (*p* < 0.001) and Meses Atrás (*p* = 0.018).

**Conclusion:**

These findings confirm the severe cognitive and executive impairments in AD patients compared to MCI and controls. The significant differences in RUDAS and INECO scores highlight their potential role in differentiating neurocognitive conditions. Impairments in control inhibitory functions and working memory were evident in AD patients. This study provides novel insights into dementia evaluation in Peru and underscores the importance of standardized neuropsychological assessments for early identification of cognitive decline. Future research should expand sample sizes and refine assessment tools to improve early detection strategies.

